# Case Report: It’s a Small Whirl Afterall

**DOI:** 10.21980/J83S8G

**Published:** 2022-01-15

**Authors:** Lisa M Schwartz, Ryan M Perdomo, Jason An

**Affiliations:** * University of California Riverside, Department of Emergency Medicine, Riverside, CA

## Abstract

Recognition of the whirl sign on computed tomography (CT) imaging can improve patient outcomes in those presenting with small bowel obstruction (SBO). In the case highlighted in this report, a 40-year-old woman with a remote history of gastric bypass presented to the emergency department (ED) with four hours of abdominal pain and vomiting. Findings on the initial CT of the abdomen and pelvis were suggestive of SBO with a whirl sign pattern. The whirl sign occurs after the bowel rotates around the mesentery, leading to a visual “whirl” of mesenteric vessels. Unfortunately, despite prompt diagnosis, the patient developed an ischemic bowel and ultimately sustained a prolonged hospital course requiring multiple bowel resections. ED providers should familiarize themselves with the whirl sign because its presence in patients with SBO increases the likelihood of ischemia. These patients should have urgent surgical consultation to decrease overall morbidity and mortality.

**Topics:**

Whirl sign, small bowel obstruction, gastric bypass, internal hernia.

[Fig f1-jetem-7-1-v5] [Fig f2-jetem-7-1-v5]

**Figure f1-jetem-7-1-v5:**
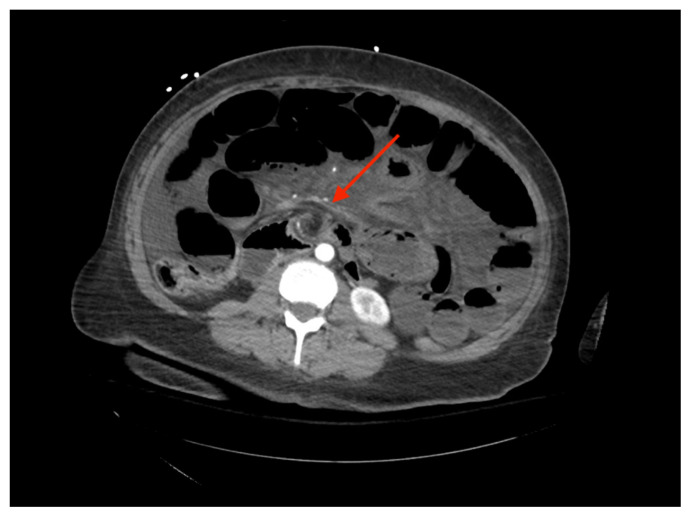


**Figure f2-jetem-7-1-v5:**
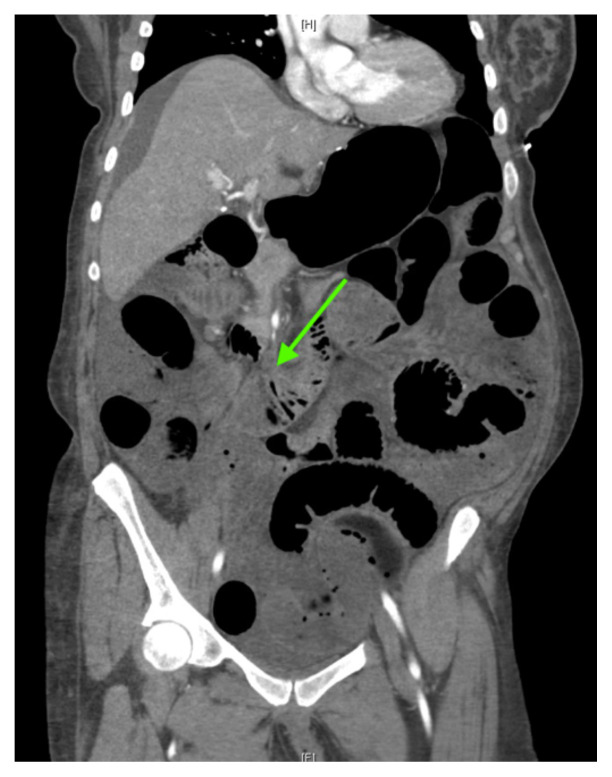


## Brief introduction

Bowel obstruction should always be considered in a clinician’s differential diagnosis for patients presenting with abdominal pain and vomiting, especially with a reported history of abdominal surgery. Bariatric surgeries have been on the rise; over 250,000 bariatric surgeries were performed in the United States in 2019.^1^ Small bowl obstruction is one important complication and can be a surgical emergency if ischemic bowel occurs, which is why it is crucial to identify it promptly. Computed tomography imaging can demonstrate compromised bowel loop as well as venous and arterial disruption, and can aid the surgeon in identifying the anatomic location of the obstruction should it require surgical intervention.^2^,^3^

This report focuses on a specific CT finding seen when the bowel vasculature is compressed, causing a “whirl” pattern, indicating a more severe obstruction if observed in a patient with SBO. The whirl sign is classically described as a closed looped pattern that is created from mesenteric soft-tissue and surrounding fat rotating around the intestinal vessels. The whirl sign should be assessed for when reading CT imaging of patients with SBO because the presence of the radiological sign and “tightness” of the whirl pattern reflects the severity of the obstruction^4^.

## Presenting concerns and clinical findings

A 40-year-old woman with a history of Roux-en-Y gastric bypass surgery (four years prior) presented to the ED with 10/10 diffuse abdominal pain. The pain started acutely four hours prior to arrival, shortly after eating. The pain was associated with nonbloody, nonbilious vomiting. On examination, the patient was ill-appearing and lethargic. She was initially tachycardic with a heart rate of 144, afebrile, and normotensive. Her abdomen was distended and diffusely tender. Imaging was delayed by several hours because she had two large bouts of hematemesis while ingesting oral contrast. She then became hypotensive and somnolent, requiring discontinuation of the CT protocol and immediate intervention in the ED. After the patient’s blood pressure was stabilized with additional crystalloid fluids and vasopressors, she underwent CT imaging. Significant labs include Hgb 12.9 gm/dL (12–16 gm/dL), WBC 13.6 k/mm^3^ (4.8–10.8 k/mm^3^), K 2.8 mmol/L (3.5–5.1 mmol/L), HCO3 14 mmol/L (21–32 mmol/L) and lactic acid 9.6 mmol/L (0.4 – 2.0 mmol/L).

## Significant findings

The CT imaging of the abdomen and pelvis demonstrated multiple loops of dilated small bowel with a whirl sign (red arrow) within the mid abdomen and a transition point (green arrow), suspicious for closed loop bowel obstruction and internal hernia.

## Patient course

The patient was resuscitated in the ED with crystalloid fluids, vasopressors, antiemetics, antibiotics, and a proton pump inhibitor. There was identification of SBO on CT imaging with a whirl pattern; the patient underwent an exploratory laparotomy within an hour of detection. In the operating room, an internal hernia was visualized with blind loop obstruction of the small bowel mesentery. Unfortunately, ischemic necrosis was identified in approximately 90% of the small bowel. The non-viable small bowel was resected and left in discontinuity. She was admitted to the intensive care unit on a ventilator with an open abdomen. Over the course of a week, she underwent two additional exploratory laparotomy procedures for pyloroplasty, appendectomy, gastric reanastomosis, gastric-tube placement, and eventual closure. The patient was eventually extubated and her diet was slowly advanced. She was discharged on postoperative day 19 from the original procedure.

Despite the prompt recognition of severe SBO by the ED provider and the urgent surgical intervention, the patient developed a significant amount of ischemic bowel prior to the exploratory laparotomy. The large quantity of small bowel that was resected ultimately resulted in short bowel syndrome, chronic diarrhea, and malnutrition.

## Discussion

The increasing number of bariatric surgeries in the United States has led to a rise in postoperative complications such as small bowel obstructions, which typically occur from adhesions or internal hernias.^5^ Bowel obstruction should always be in the clinician’s differential diagnoses when treating a patient with prior intra-abdominal surgical history who presents with abdominal pain or vomiting.

Oftentimes SBOs may be managed medically, which avoids the complications associated with a surgical laparotomy. However, SBO with components of strangulation typically requires surgical management and therefore should not be missed.^6^ When surgery is indicated, delays have been shown to increase morbidity and mortality.^7^

One should not only interpret CT imaging in real time but also be able to identify and understand the clinical significance of a whirl sign, if present. Patients diagnosed with SBO who had the presence of a whirl pattern on CT were over 25 times as likely to require surgical intervention.^8^ A retrospective study of 194 patients found a high negative predictive value of 86% and high positive predictive value of 80% of SBO necessitating surgery.^8^ This study also showed a high specificity of 94%. Thus, the identification of the whirl sign on a patient with abdominal complaints should raise suspicion not only for an SBO, but also should indicate the clinical severity of the obstruction.

The whirl sign does have its limitations since it may also be seen in other cases including volvulus, omental torsion, and enteritis. In addition, multiple factors can limit the image reliability such as missed diagnostic identification of the whirl sign, or the CT omission of the whirl pattern due to size and/or perpendicular slicing of the CT cut.^9^ Therefore, documented sensitivity of the sign is only 60%^8^. Finally, although the whirl sign significantly increases the chances the patient will require surgical intervention, there are still patients that can be successfully treated with conservative medical management.

The learning point of this case is the importance of timely recognition and management of patients with severe SBO to reduce morbidity and mortality. One should evaluate for the presence of a whirl sign in patients with SBO because it can indicate bowel ischemia that may necessitate urgent surgical intervention.

## Supplementary Information






