# Supplementary figures and images for: Case Report: It’s a Small Whirl Afterall
Source: J Educ Teach Emerg Med. 2022 Jan 15;7(1):V5–7. doi: 10.21980/J83S8G (PMC10358867; doi:10.21980/J83S8G)

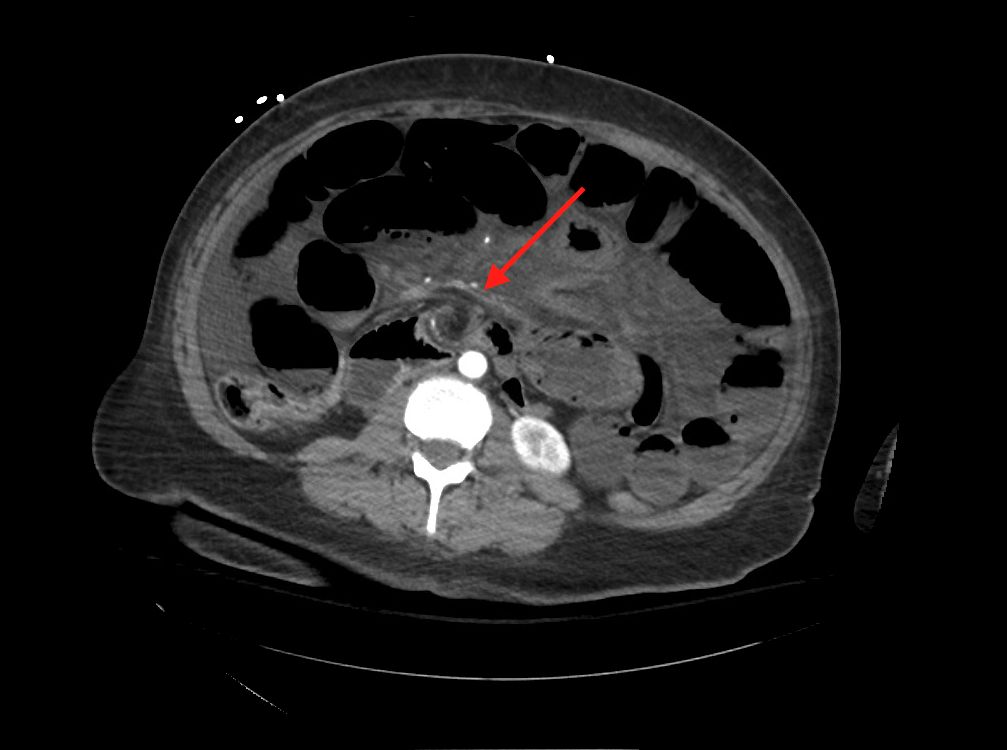

Supplement: Supplementary file 1 [file JETem-7-1-V5-supp1.jpeg]

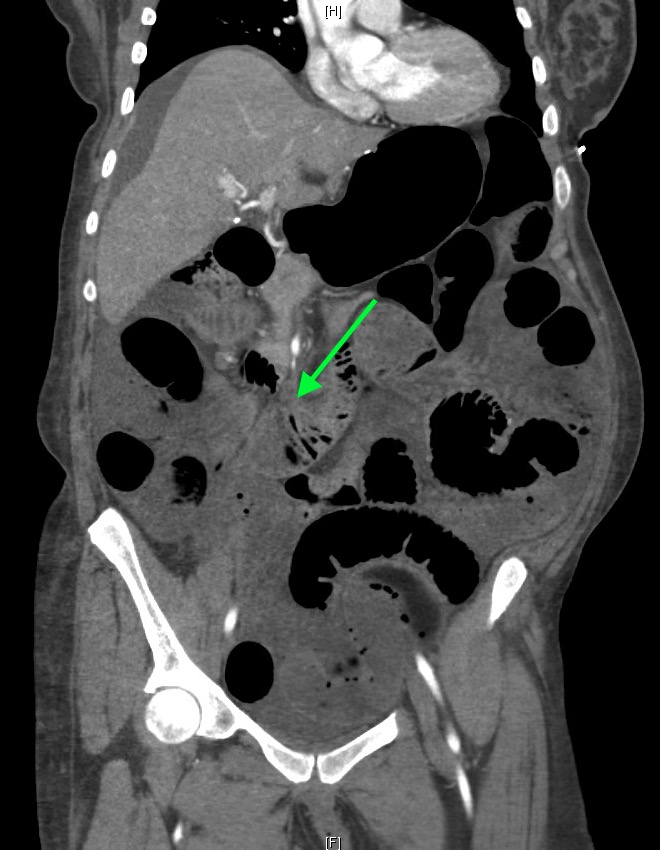

Supplement: Supplementary file 2 [file JETem-7-1-V5-supp2.jpeg]
